# Ascites-derived hsa-miR-181a-5p serves as a prognostic marker for gastric cancer-associated malignant ascites

**DOI:** 10.1186/s12864-024-10359-2

**Published:** 2024-06-24

**Authors:** Yongchao Yang, Junliang Zhang

**Affiliations:** 1 Department of General Surgery 1, Sunshine Union Hospital, Weifang City, 261072 Shandong Province China; 2 Department of Emergency Medicine, Sunshine Union Hospital, No. 9000, Yingqian Street, High-tech Zone, Weifang City, 261072 Shandong Province China

**Keywords:** miRNAs, Gastric cancer, Ascites samples, Hsa-miR-181b-5p, Survival

## Abstract

**Background:**

Peritoneal carcinomatosis was the main reason leading to gastric cancer (GC)-related death. We aimed to explore the roles of dysregulated microRNAs (miRNAs) and related immune regulation activities in GC-associated malignant ascites.

**Methods:**

GSE126399 were downloaded from GEO database. Differentially expressed miRNAs in GC ascites samples was firstly screened, and critical miRNAs were further investigated by LASSO (least absolute shrinkage and selection operator) logistic regression and random forest (RF) algorithm. Receiver operating characteristic of critical miRNAs was also constructed. Moreover, functional analysis, immune cell infiltration associated with differentially expressed mRNAs were further analyzed. After selecting key modules by weighted gene co-expression network analysis, mRNAs related with survival performance and transcription factor (TF)-miRNA-mRNA network were constructed.

**Results:**

Hsa-miR-181b-5p was confirmed as critical differentially expressed miRNAs in GC ascites. Then, the tumor samples were divided into high- and low- expression groups divided by mean expression levels of hsa-miR-181b-5p, and subjects with high hsa-miR-181b-5p levels had better survival outcomes. In total, 197 differentially expressed mRNAs associated with hsa-miR-181b-5p levels were obtained, and these mRNAs were mainly enriched in muscle activity and vascular smooth muscle contraction. Hsa-miR-181b-5 was positively related with activated CD4 T cells and negatively related with eosinophil. 17 mRNAs were selected as mRNAs significantly related with prognosis of GC, such as PDK4 and RAMP1. Finally, 75 TF-miRNA-mRNA relationships were obtained, including 15 TFs, hsa-miR-181b-5p, and five mRNAs.

**Conclusion:**

Our data suggest that the differentially expressed hsa-miR-181b-5p in ascites samples of GC patients may be a valuable prognostic marker and a potential target for therapeutic intervention, which should be validated in the near future.

**Supplementary Information:**

The online version contains supplementary material available at 10.1186/s12864-024-10359-2.

## Background

Gastric cancer (GC) is a heterogeneous and complicated disease with the characterization of primary stomach epithelial malignancy and responsible for 0.769 million deaths in 2020 globally [1]. The disease ranks as the fifth most common malignancy globally and the third mortality and incidence in China. Peritoneal carcinomatosis, as a terminal condition, was recognized as the reason for leading to 60% of all the deaths from GC. Meanwhile, the condition was usually related with ascites accumulation, which caused the poor prognosis and reduced quality of life [2]. According to the complicated pathogenesis, more attention should be paid for the better surveillance of the treatment strategy.

Malignant cells from primary tumor’s serosal surface could be detached to the peritoneal surface, which lead to more complex disease condition. Until to now, various of factors related with GC prognosis have been reported, such as depth of invasion, histological type, tumor location, age, and gender [3]. As the development of molecular biological technologies, multiple studies have been designed to explore molecular biomarkers related with GC development. For example, Li et al. showed that 2371 mRNAs and 350 long non-coding RNAs (lncRNAs) with differentially expressed levels in GC [4]. Moreover, tumor markers in blood were widely investigated in GC patients, such as CA 125, CA 19 − 9, carcinoembryonic antigen [5, 6]. Currently, accurate diagnosis of GC is based on the cytological analysis of ascites. Thus, it is inferred that the tumor makers in ascites may elicit promising predictive performance in GC.

Accumulating evidence has demonstrated that microRNAs (miRNAs), as the small non-coding RNAs play critical role in various cancers, including GC [7, 8]. However, the role of miRNAs in ascites has not been elucidated fully. Therefore, in this study, we retrieved GSE126399 dataset from GEO database, and identified the differentially expressed miRNAs in ascites of GC patients. The key miRNAs were screened by machine learning methods. The differentially expressed mRNAs between high and low miRNA expression group were analyzed based on TCGA (The Cancer Genome Atlas) -STAD (stomach adenocarcinomas) dataset, followed by function enrichment analysis, immune infiltration analysis, weighted gene co expression network analysis (WGCNA) and transcription factor (TF)-miRNA-mRNA network construction. The flow chart of this study is illustrated in Supplementary Fig. 1. In the present study, we aimed to explore the ascites-derived miRNA biomarker for the diagnosis and prognosis of GC.

## Methods

### Data source

GSE126399 was downloaded from Gene expression omnibus (GEO) database (https://www.ncbi.nlm.nih.gov/geo/), which included 10 liver cirrhosis-associated benign ascites and 12 malignant ascites from GC. Controls showed matched age, gender and gender with GC patients (Supplemental Table 1). The data were sequenced based on GPL18402 Agilent-046064 Unrestricted_Human_miRNA_V19.0_Microarray.

Gene RNA sequencing (RNAseq) expression matrix data of stomach adenocarcinoma (STAD), as well as survival information, and clinical information were all obtained from the University of California Santa Cruz (UCSC) database (https://xena.ucsc.edu/welcome-to-ucsc-xena/). Totally, 375 tumor samples and 32 normal samples were involved in the mRNA expression matrix, and 434 tumor samples and 41 normal samples were involved in the microRNA (miRNA) expression matrix. The patients and controls matched on age and gender in TCGA cohorts (Supplemental Tables 2 and Table 3). Among them, 405 tumor samples supplemented with additional information including clinical and survival data were obtained.

### Screening differentially expressed miRNAs

Differentially expressed miRNAs in GC were screened using “limma” in R package [9]. Then, the results were visualized using ggplot2 in R package [10]. *P* < 0.05 and |log_2_ fold change (FC)| >0.5 were defined as thresholds for screening differentially expressed miRNAs.

### Prediction of critical miRNAs in GC-associated ascites

LASSO (least absolute shrinkage and selection operator) logistic regression was designed to predict sample classification based on expression values of differentially expressed miRNAs in each sample in the GSE126399 dataset combined with the grouping information of samples [11]. Ten-fold cross validation was performed to reduce feature dimensions using R software “glmnet” package (version 4.0–2), and the parameters were set as follows: family="binary”, type. measure="class”, nfold = 10. The error rate under different features were calculated using ten-fold cross validation and selected strong correlation features. Furthermore, the error graph of cross validation and the graph of gene coefficient were both constructed to screen critical differentially expressed miRNAs.

Random forest (RF) is a compositional supervised learning method and an extension of decision tree [12]. The prediction model was constructed via RF by classifying objects and using multiple decision trees. Finally, the classification results of each decision tree were summarized. RF algorithm was performed using random forest method in the R package randomForest to screen critical miRNAs related with GC [13]. Subsequently, the critical miRNAs were sorted by the RF algorithm according to “Mean DecreaseAccuracy” and “Mean DecreaseGini” respectively.

Finally, critical miRNAs were selected by intersecting the critical miRNAs selected by LASSO logistic regression algorithm and RF algorithm.

### Diagnostic ability analysis of critical miRNAs in GC-associated ascites

In order to evaluate the diagnostic value of critical miRNAs, the R package “pROC” was used to construct receiver operating characteristic (ROC) of critical miRNAs in GSE126399 [14].

### Differentially expressed miRNAs in GC-associated ascites and its relationship with clinical features

In order to clarify the correlation between critical miRNAs levels and clinical characteristics, the clinical information of included subjects was selected, including age, gender, neoplasm_histologic_grade, pathologic_M, pathologic_N, pathologic_T, tumor_stage, and overall survival in the dataset TCGA -STAD.

Combined with the levels of critical differentially expressed miRNAs, the clinical characteristics significantly different between the high and low expression groups were screened through Chi-squared test. Clustering heatmaps of clinical features in patients with different critical miRNAs levels was visualized using R package “ComplexHetmap”.

### Differentially expressed mRNA selection and functional analysis

The subjects were divided into high- and low- expressed groups by the levels of critical differentially expressed miRNAs. Subsequently, limma was used to screen mRNAs with different levels in TCGA-STAD between high- and low- expressed groups [9]. Multiple test correction was further performed using the Benjamin&Hochberg method. The threshold for selecting differentially expressed mRNA was set as follows: adj. *P*.Value < 0.05 and |log_2_FC (fold change)|>1.

The Gene Ontology (GO) system includes biological process (BP), molecular functions (MF), and cellular components (CC) [15]. Functional enrichment analysis, including GO and Kyoto Encyclopedia of Genes and Genomes (KEGG) pathway were both performed to screen the potential function of different expressed mRNAs, and the enrichment threshold was set as *P* < 0.05 and enrichment count ≥ 2.

### Immune cell infiltration

To investigate the status of immune cell associated with critical miRNA levels, the abundance of 28 types of immune cell infiltration in the high and low expression group samples was calculated using ssGSEA (single sample gene set enrichment analysis) algorithm based on GSVA (gene set variation analysis) in R package [16]. To further screen out immune cells with different infiltration levels between high and low expression groups, a box plot was drawn using the R package “ggplot2” based on the wilcox.test test. Then, the spearman method was used to calculate the correlation between the levels of critical miRNAs and the infiltration levels of immune cells. The correlation lollipop map was visualized using ggplot2 in R package.

### Key modules selected by WGCNA

WGCNA is a tool for identifying gene expression patterns of multiple samples [17], which can analyze the association between the module and specific traits or phenotypes and cluster genes with similar expression patterns. Therefore, it is widely used in the research of disease and gene association analysis.

In order to find genes highly related with clinical characteristics, WGCNA was performed using WGCNA in R package. The samples were clustered, and the soft threshold of the data was determined. In order to screen out key modules related to sample traits, *p* < 0.05 and correlation coefficient > 0.3 were defined as thresholds for selecting modules. The modules closely related with six types of immune cells at least with differential distribution were set as key modules.

Subsequently, the intersection of mRNAs involved in key modules and differentially expressed mRNAs was selected using VennDiagram in R package [18].

### mRNAs related with survival performance

High and low expression groups were divided by the median expression value of intersection miRNA. Kaplan-Meier (K-M) survival analysis was performed on the high and low expression groups using R-package survival to screen out key mRNAs related to prognosis [19]. Pearson correlation between critical miRNAs and mRNAs was calculated. Finally, the results were visualized using ggplot2 in R-package.

### Transcription factors (TF)-miRNA-mRNA network

The mRNAs-miRNAs pairs were investigated by tools of miRwalk3.0 [20] and ENCORI (The encyclopedia of RNA interactomes, https://starbase.sysu.edu.cn/index.php). The thresholds for miRwalk3.0 was set as follows: binding probability ≥ 0.95; binding site position: 3UTR. The thresholds for ENCORI was set as follows: CLIP-Data ≥ 1, Degradome-Data ≥ 0, pan-Cancer ≥ 0, programNum ≥ 1. Finally, miRNA-mRNA interaction pairs of the two databases were selected.

TFs of miRNAs were further explored using the tool of TransmiR v2.0 (http://www.cuilab.cn/transmir). Finally, TF-miRNA-mRNA network was constructed using Cytocape software.

## Results

### Differentially expressed miRNA in GC- associated malignant ascites

**Fig. 1:**
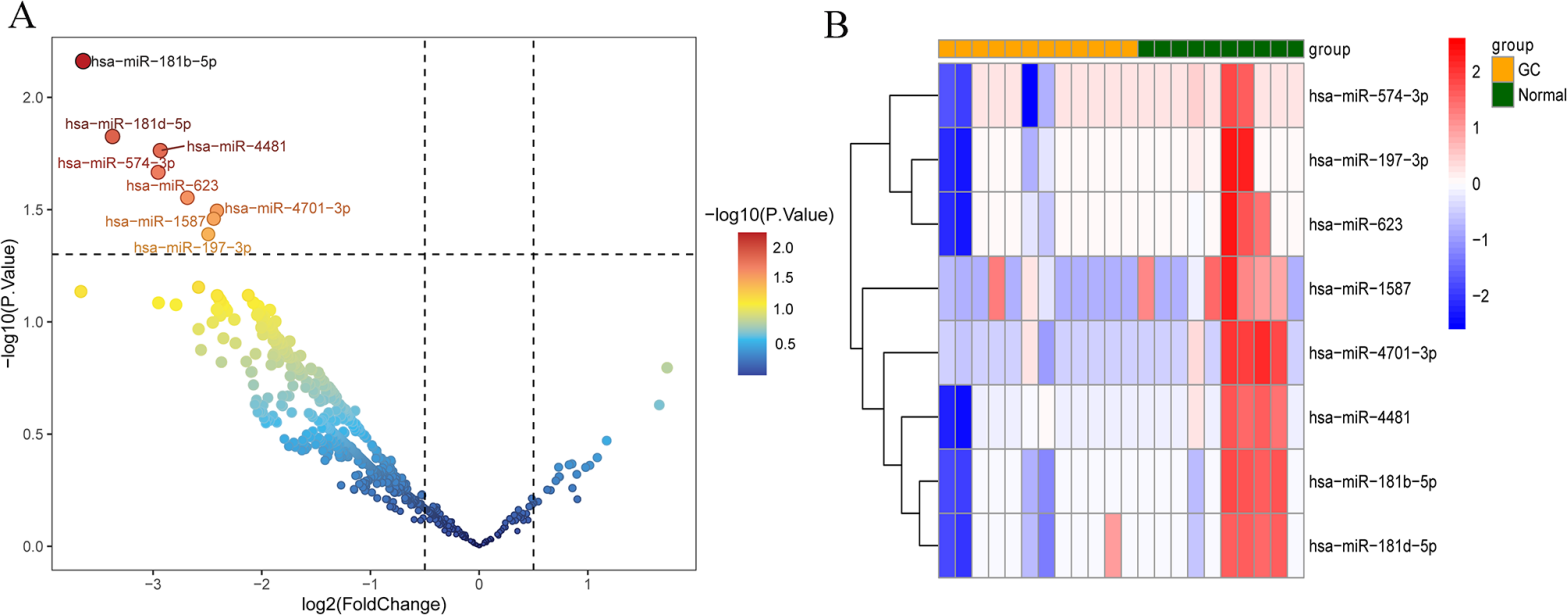
Volcano plot and heatmap of differentially expressed miRNA between gastric cancer and normal controls. **A**: volcano plot of differentially expressed miRNA in gastric cancer; **B**: heatmap of differentially expressed miRNA in gastric cancer. Each small square represents each miRNA, and its color represents the expression level of miRNA, and the darker color represents the higher expression level

Volcano plot of differentially expressed miRNA in GC-associated malignant ascites was shown in Fig. 1A, which revealed that eight miRNAs had significantly lower levels in GC, including has-miR-574-3p, has-miR-197-3p, has-miR-623, has-miR-1587, has-miR-4701-3p, has-miR-4481, has-miR-181b-5p, and has-miR-181d-5p. Furthermore, Fig. 1B shows that differentially expressed miRNAs in GC-associated malignant ascites and liver cirrhosis-associated benign ascites has obviously different expression levels.

### Critical miRNAs based on machine learning

**Fig. 2:**
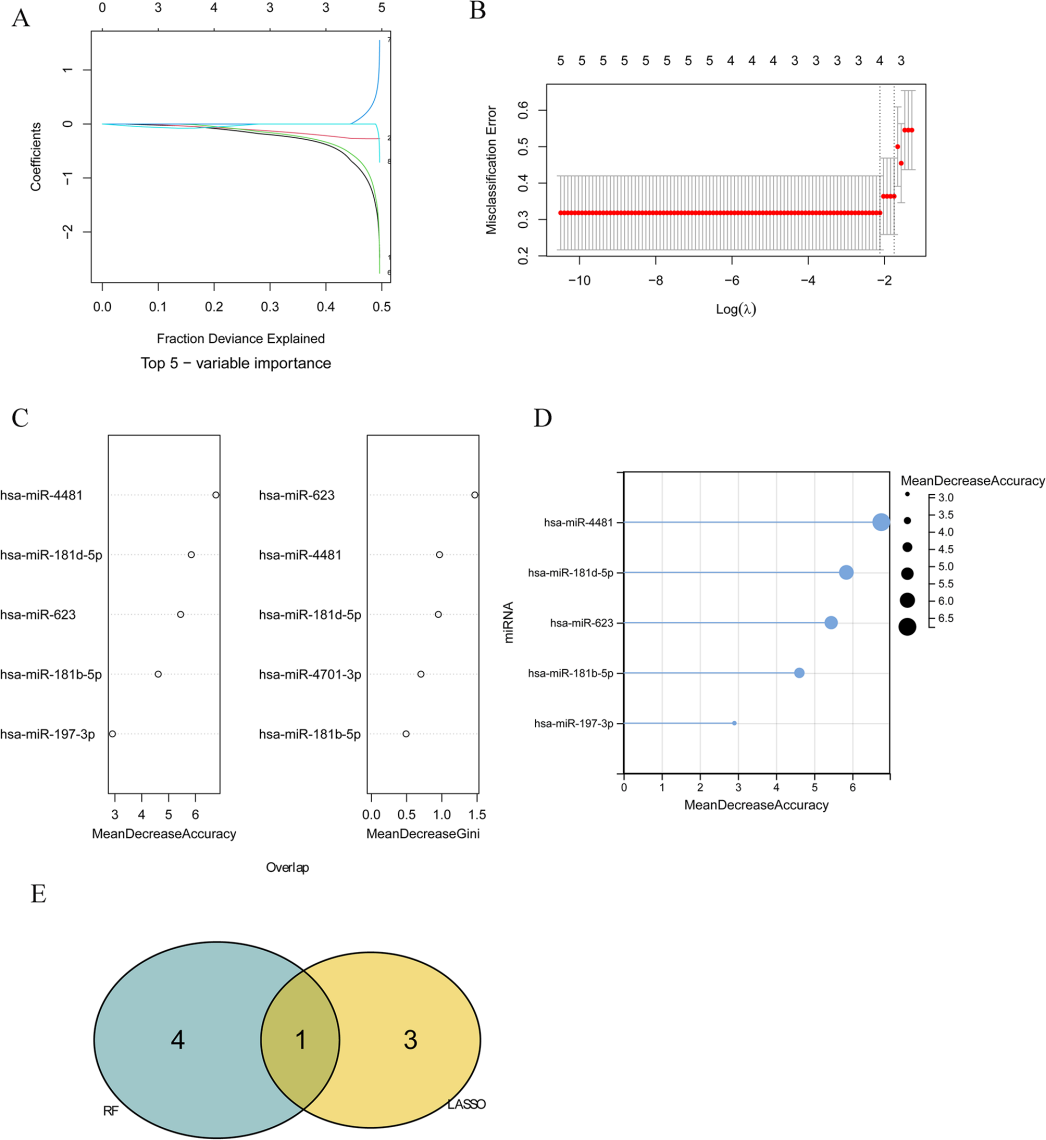
The critical miRNAs selected by machine learning algorithms. **A**: gene coefficient variation graph in LASSO model. Each curve in the graph represents the variation trajectory of each independent variable coefficient, with the y-axis representing the coefficient value and the upper x-axis representing the number of non-zero coefficients in the model; **B**: penalty graph of LASSO logic coefficient; **C**: top 5 critical miRNAs. “Mean Decrease Accuracy” and “Mean Decrease Gini” are two important indicators in random forest models. Among them, “mean Decrease accuracy “indicates the degree of decrease in the accuracy of random forest prediction, and the larger value represents the greater importance of the variable; Mean decrease Gini “calculates the impact of each variable on the heterogeneity of observations at each node of the classification tree to compare the importance of the variables. The higher value represents greater importance of variables. **D**: Lollipop chart of top 5 miRNAs. **E**: Venn diagram of critical miRNAs

Critical miRNAs were firstly selected by LASSO logistic regression analysis. The graph of gene coefficients (Fig. 2A) and the error plot of cross validation (Fig. 2B) were both constructed. Minimum error rate reached the lowest when lambda. min was 0.1200739. Four critical miRNAs were selected by LASSO logistic regression analysis, including hsa-miR-4701-3p, hsa-miR-1587, hsa-miR-574-3p, and hsa-miR-181b-5p.

Meanwhile, critical miRNAs were also selected by RF model analysis. The selected miRNAs were sorted by “Mean DecreaseAccuracy” and “Mean Decrease Gini” (Fig. 2C). The top five miRNAs were selected, including hsa-miR-4481, hsa-miR-181d-5p, hsa-miR-623, hsa-miR-181b-5p, and hsa-miR-197-3p (Fig. 2D).

Finally, hsa-miR-181b-5p was confirmed as the only one intersection of LASSO algorithm and RF algorithm (Fig. 2E).

### The diagnostic and prognostic role of hsa-miR-181b-5p

**Fig. 3:**
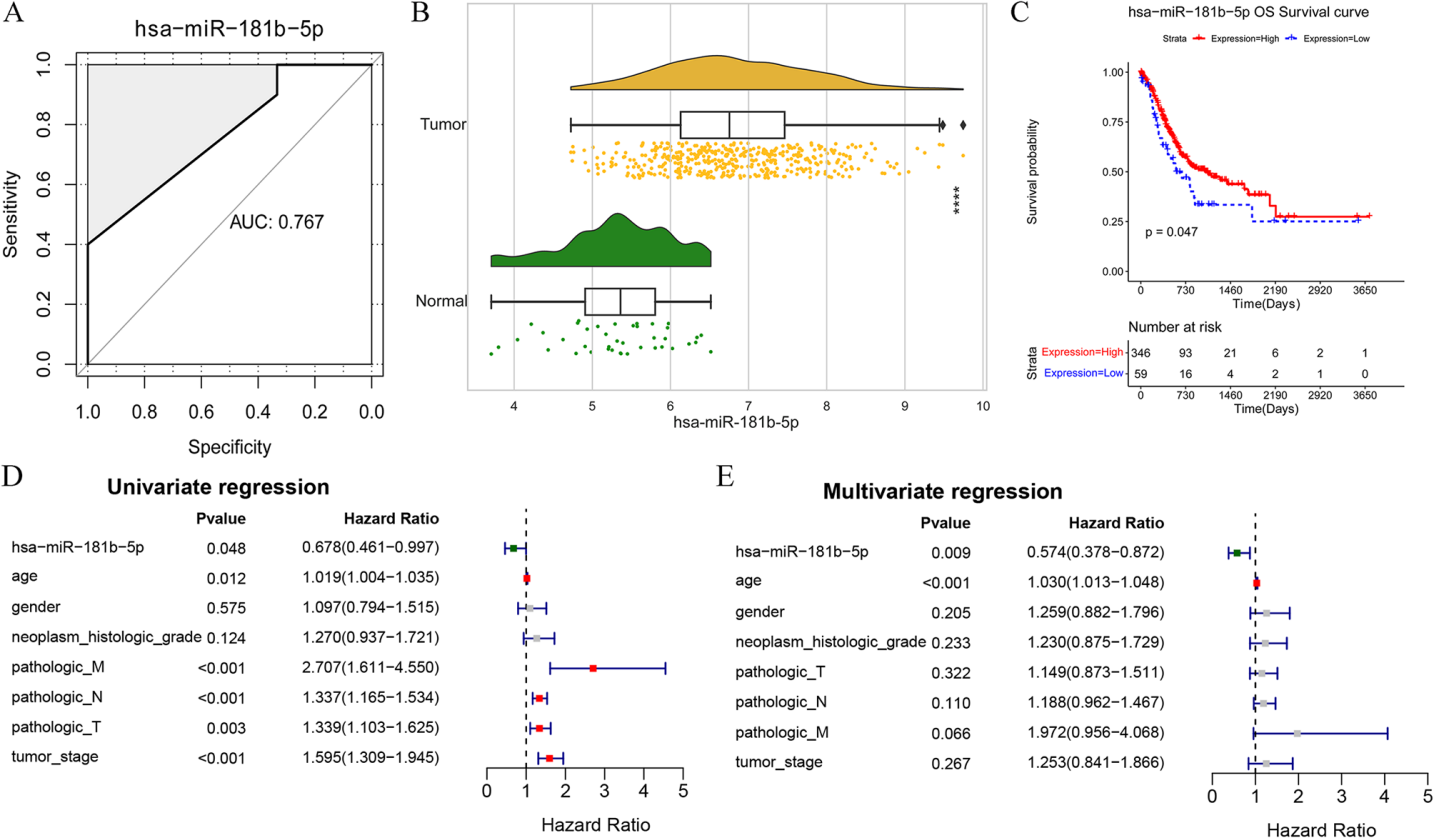
Evaluation of diagnostic value of critical miRNAs. **A**: receiver operating characteristic (ROC) curve of hsa-miR-181b-5p; **B**: Expressing cloud and Rain maps of hsa-miR-181b-5p in tumor and controls. * versus controls, *p < 0.05, **p < 0.01, *** *p* < 0.001, **** *p* < 0.0001; **C**: Kaplan-Meier curve of hsa-miR-181b-5p. **D**: univariate regression analysis; **E**: multivariate regression analysis

As shown in Fig. 3A, the AUC of hsa-miR-181b-5p for diagnosing GC was 0.767, suggesting the good predictive ability of hsa-miR-181b-5p for identifying GC from controls.

Expressing cloud and rain maps of hsa-miR-181b-5p showed the expression level of hsa-miR-181b-5p in tumor was significantly higher than that in normal controls (Fig. 3B). Furthermore, based on R Package Surveyor, the optimal threshold of miRNA was calculated as 5.868633. Then, the tumor samples were divided into high and low expressed groups based on the optimal threshold. Then, K-M curves of the high and low expressed groups were analyzed based on logrank test (Fig. 3C). Subjects with high expressed levels of hsa-miR-181b-5p had better survival outcomes (*P* = 0.047). Furthermore, univariate and multivariate regression analyses were performed for hsa-miR-181b-5p and various clinical features. Results indicated that hsa-miR-181b-5p was an independent prognostic factor (all *p* < 0.05, Fig. 3D and E).

### The relationship between miRNAs and clinical characteristics

The effect of hsa-miR-181b-5p on clinical characteristics was explored, including age, gender, neoplasm-histologic-grade, pathologic-M, pathologic-N, pathologic-T, tumor stage, and OS. The comparison of clinical characteristics between high and low expression group was performed in Table 1. Notably, OS could be significantly affected by the level of hsa-miR-181b-5p (*P* = 0.04016).

**Table 1:** The comparison of clinical characteristics between high and low expression groups

features	High.exp (hsa-miR-181b-5p, *n* = 346)	Low.exp (hsa-miR-181b-5p, *n* = 59)	*p*value
**OS**			0.04016
Alive	211	27	
Dead	135	32	
**age**			0.0531
< 60	101	25	
≥ 60	243	33	
**gender**			0.5624
female	118	23	
male	228	36	
**neoplasm_histologic** **_grade**			0.8232
G1	7	1	
G2	124	18	
G3	209	37	
**pathologic_M**			0.7811
M0	308	54	
M1	23	3	
**pathologic_N**			0.7714
N0	102	19	
N1	92	18	
N2	69	10	
N3	74	10	
**pathologic_T**			0.1402
T1	15	4	
T2	68	18	
T3	162	21	
T4	98	14	
**tumor_stage**			0.1089
stage i	38	13	
stage ii	110	17	
stage iii	151	22	
stage iv	34	4	

### Differentially expressed mRNAs in high and low expression groups

**Fig. 4:**
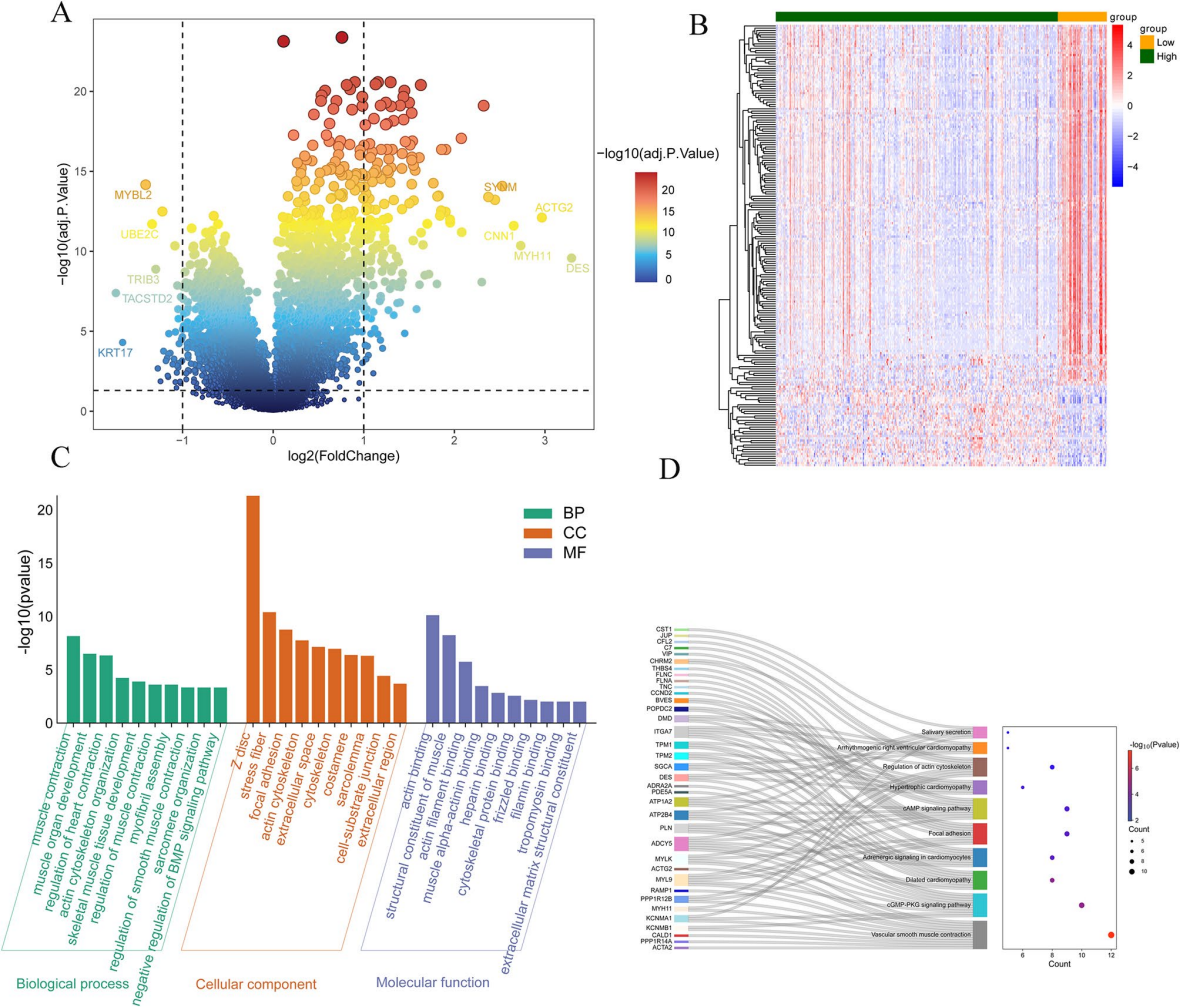
Differentially expressed mRNA between high- and low-expression groups and its functional enrichment. **A**: volcano plot of differentially expressed mRNA in gastric cancer; **B**: heatmap of differentially expressed mRNA in gastric cancer. Each small square represents each miRNA, and its color represents the expression level of miRNA, and the darker color represents the higher expression level; **C**: top 10 functional GO items enriched by differentially expressed mRNA; **D**: Top 10 KEGG pathways enriched by differentially expressed mRNA. The left side represents the differential mRNA enriched by the pathway, the right side represents the top 10 pathway, and the horizontal axis in the bubble diagram on the right side represents the number of differential mRNA enriched by the pathway. The larger the bubble represents the more enriched the differential mRNA. The color of the bubble represents significant *p* value, and the redder represents the smaller *p* value

In total, 197 differentially expressed mRNAs were related with miRNAs levels, including 161 up-regulated mRNAs and 36 down-regulated mRNAs. Furthermore, volcano plot (Fig. 4A) and heatmap (Fig. 4B) showed the relative levels of mRNAs in high and low expression groups.

### Functional enrichment analysis

Top 10 GO items were shown in Fig. 4C, including muscle contraction, muscle organ development, regulation of heart contraction. Meanwhile, top 10 enriched KEGG pathways were shown in Fig. 4D, including vascular smooth muscle contraction, cGMP-PKG signaling pathway, and dilated cardiomyopathy.

### Immune microenvironment associated with hsa-miR-181b-5p

**Fig. 5:**
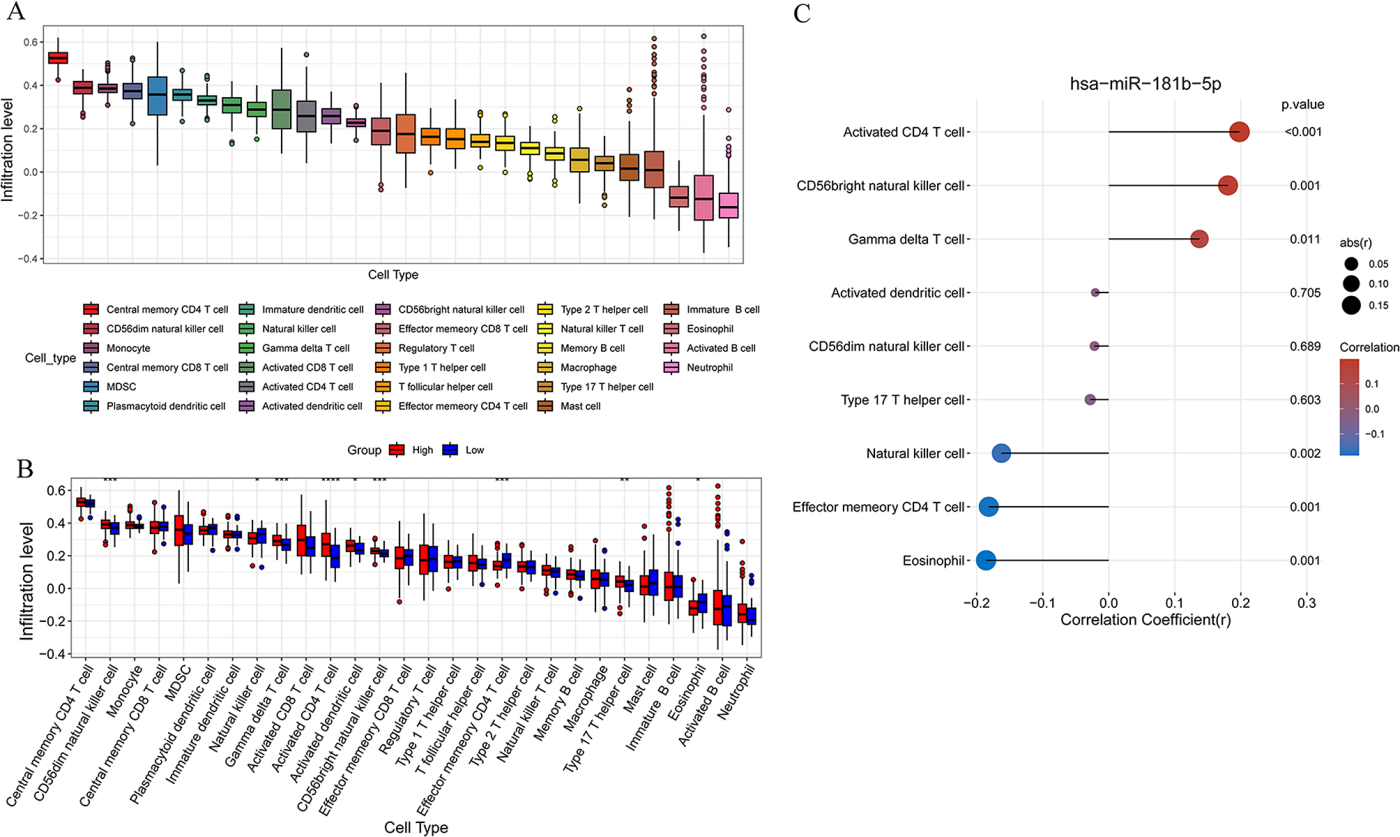
Immune microenvironment analysis of gastric cancer. **A**: boxplot of 28 types of immune cell infiltration in TCGA-STAD; **B**: comparison of 28 types of immune cell infiltration between subjects in high- and low-expression groups. * versus controls, *p < 0.05, **p < 0.01, ***p < 0.001, ****p < 0.0001; **C**: Correlation lollipop chart of hsa-miR-181b-5p and immune cells with different infiltration levels

The abundance of 28 immune cell infiltration of samples in TCGA-STAD was shown in Fig. 5A. Furthermore, the infiltration of 28 immune cells in high and low expression groups was compared, and the infiltration of nine immune cells had significant difference between high and low expression groups, including activated CD4 T cell, activated dendritic cell, CD56 bright natural killer cell, CD56 dim natural killer cell, effector memory CD4 T cell, eosinophil, gamma delta T cell, natural killer cell, and type 17 T helper cell (Fig. 5B). Then, the association between hsa-miR-181b-5 and nine immune cells was investigated (Fig. 5C), and hsa-miR-181b-5 was positively related with activated CD4 T cells and negatively related with eosinophil.

### WGCNA analysis

**Fig. 6:**
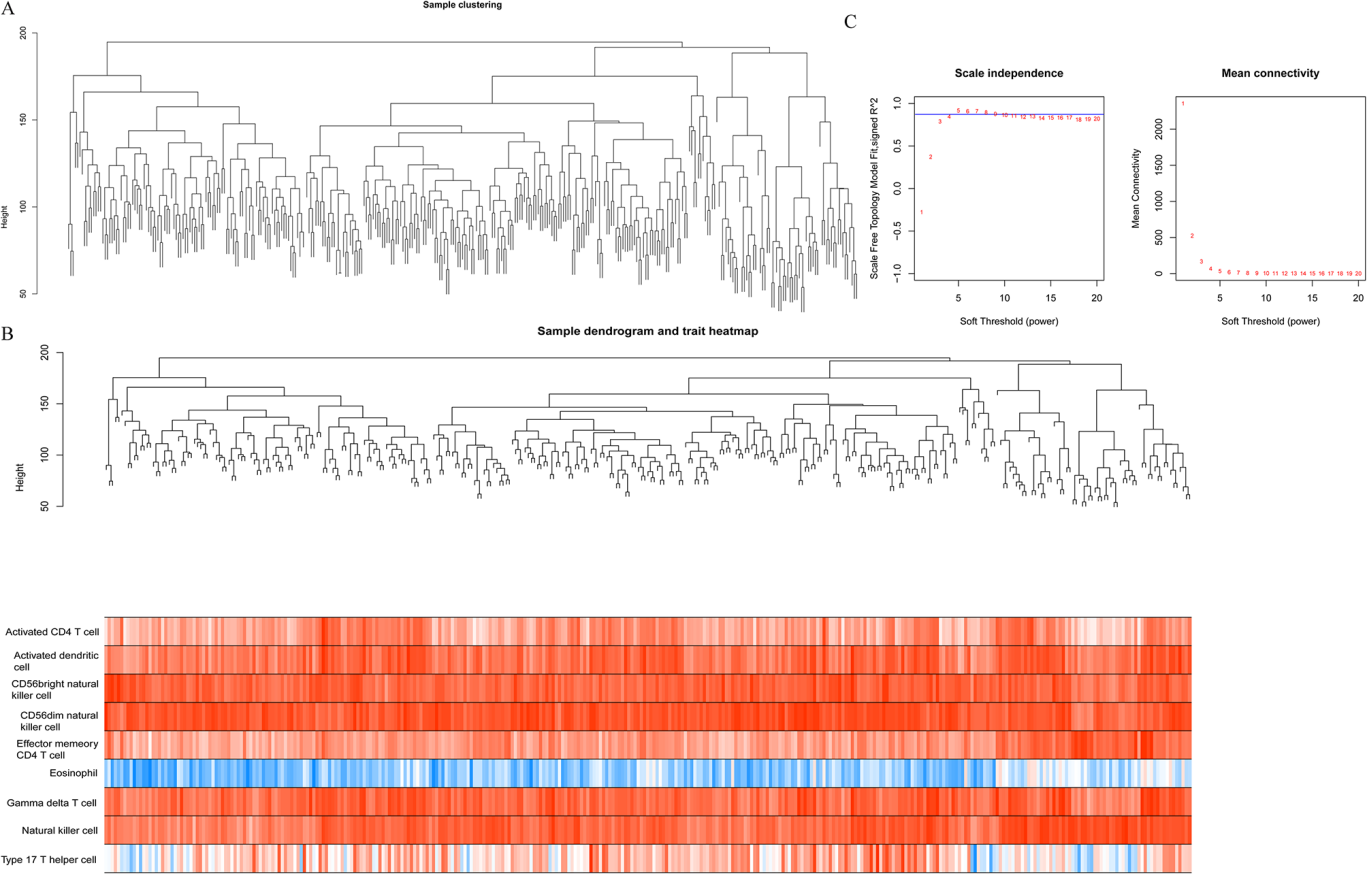
Sample clustering and soft threshold determination by weighted gene co-expression network analysis (WGCNA). **A**: sample clustering of TCGA-STAD; **B**: data sample clustering and phenotypic information. The upper half shows the sample clustering situation, and the lower half shows nine immune cell traits; **C**: Scale free soft threshold distribution. The horizontal axis represents the weight parameter power value. The vertical axis in the left image represents the Scale Free Topology Model Fit, that is, signed R ^ 2. The square of the correlation coefficient reaches 0.85 or more, indicating that the network is approaching a scale-free distribution. The vertical axis in the right image represents the average value of all gene adjacency functions in the corresponding gene module

As shown in Fig. 6A, the overall clustering of the dataset samples achieves good performance, so we did not exclude the samples. Subsequently, the characteristics of the samples were sorted out and added to the clustering graph to construct the sample clustering and clinical trait heatmap (Fig. 6B). When power was selected as 5, the value of signed R^2 was more than 0.85, suggesting the network was close to scale-free networks. Meanwhile, the mean of the adjacency function in the middle was gradually approaching 0 with a gentle trend (Fig. 6C).

### Co-expression network and their association with immune cells

**Fig. 7:**
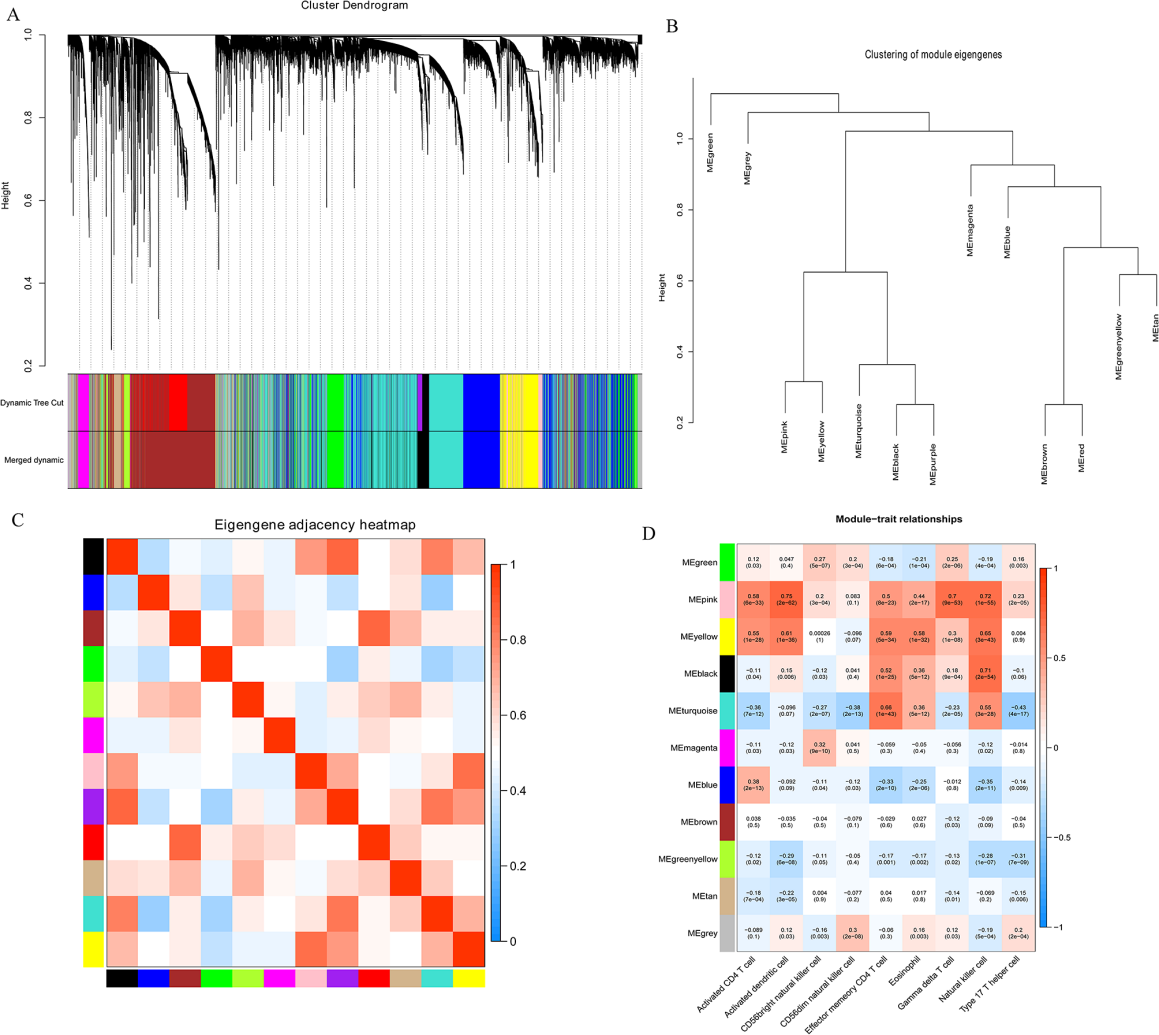
Co-expression network. **A**: module clustering tree. Genes are divided into various modules through hierarchical clustering, with different colors representing different modules; **B**: cluster tree of modules; **C**: heatmap of modules; **D**: heatmap of module and clinical trait. The vertical axis represents different modules, and the horizontal axis represents different traits. Each block represents the correlation coefficient and significance *P*-value between a certain module and a certain trait

The minimum number of genes for each gene module was set as 70 under the standard hybrid dynamic tree cutting algorithm. Then, a total of 13 modules were obtained. To merge similar modules based on the results of the dynamic pruning tree algorithm, MEDisThres was set as 0.3. After merging, a total of 11 modules were clustered (Fig. 7A).

The cluster tree of modules was shown in Fig. 7B. Meanwhile, the association of the 11 modules was shown in the heatmap (Fig. 7C). Moreover, MEpink, MEyellow, and MEturquoise were significantly related with most of immune cells (Fig. 7D). In MEpink, 649 genes were involved, 1743 genes were included in MEyellow, and 5472 genes were involved in MEturquoise. Totally, 7864 genes were defined as hub genes.

### mRNAs related with immune cells

**Fig. 8:**
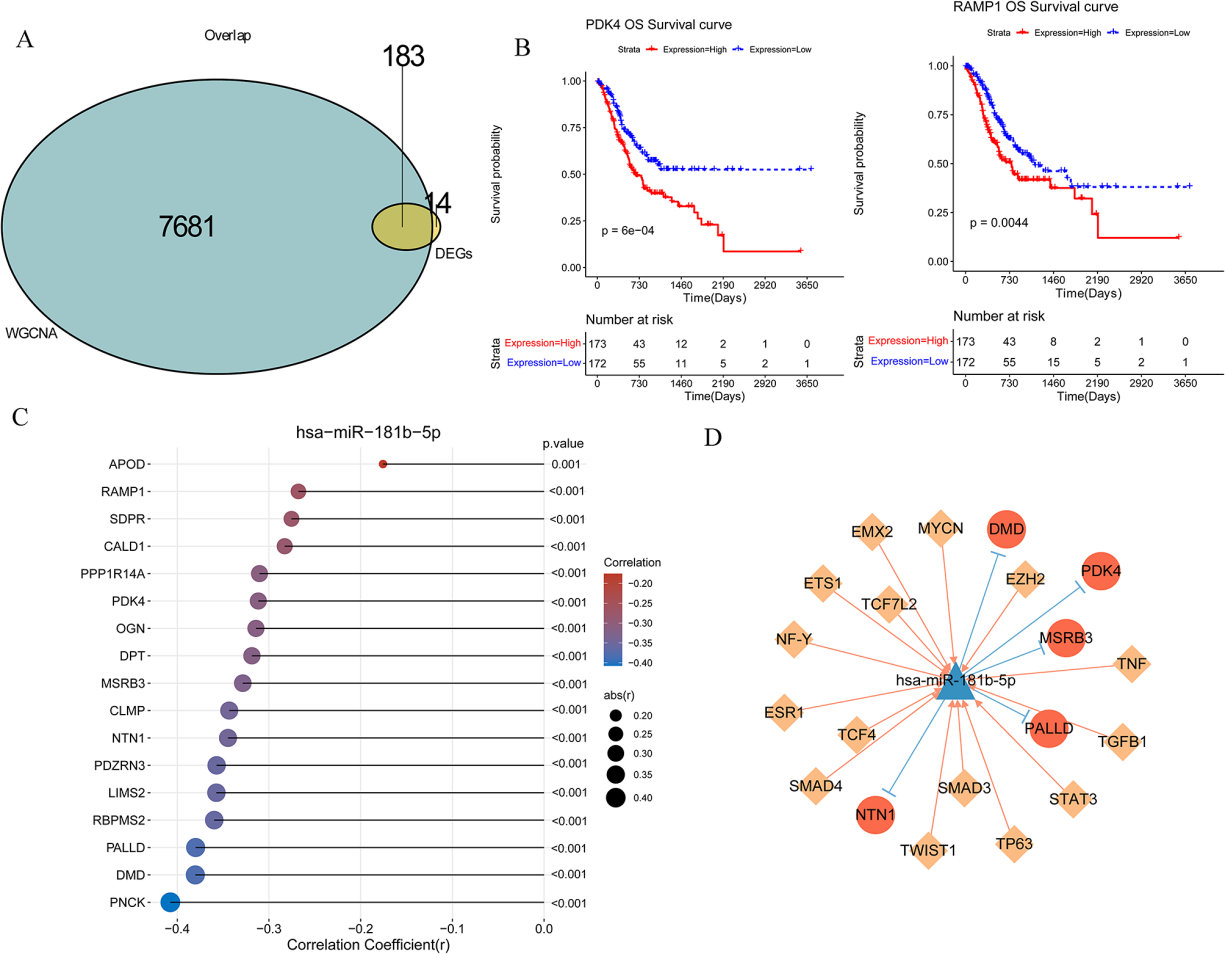
MRNAs related with immune roles, Kaplan-Meier survival analysis, and transcription factor (TF)-miRNA-mRNA regulation network. **A**: Venn diagram of intersecting genes; **B**: Kaplan-Meier curve of PDK4 and RAMP1; **C**: lollipop chart of critical mRNAs and hsa-miR-181b-5p. **D**: The red circle represents mRNA; Yellow diamond represents TF; The blue triangle represents miRNA; The pink arrow line represents TF competitive binding miRNA, and the blue T-shaped line represents miRNA mRNA regulatory relationship

In order to select mRNAs related with immune cells, 7681 hub genes and differentially expressed mRNAs were taken the intersection. Finally, 183 mRNAs were recognized as mRNAs related with immune cells (Fig. 8A).

The prognosis role of immune cells-related mRNAs.

In total, 17 mRNAs were selected as mRNAs significantly related with prognosis of GC (Table 2). Then, K-M curves based on expression levels of PDK4 and RAMP1 were constructed, respectively (Fig. 8B). The curves showed that low levels of PDK4 and RAMP1 had better overall survival time.

**Table 2:** mRNAs related with prognostic of gastric cancer

mRNA	*p*value	High.number	Low.number
PDK4	0.000595952	173	172
RAMP1	0.004402854	173	172
APOD	0.01527292	173	172
MSRB3	0.016481641	173	172
PDZRN3	0.020938397	173	172
NTN1	0.023905938	173	172
CALD1	0.024431804	173	172
OGN	0.025474369	173	172
DPT	0.02744154	173	172
PPP1R14A	0.027695212	173	172
CLMP	0.02900008	173	172
PNCK	0.036473881	173	172
LIMS2	0.038730822	173	172
DMD	0.042339273	173	172
RBPMS2	0.043804455	173	172
PALLD	0.044257273	173	172
SDPR	0.048025518	173	172

Then, the association between hsa-miR-181b-5p and selected mRNAs was calculated, and we found that hsa-miR-181b-5p was negatively related with all 17 mRNAs (Fig. 8C). Among them, hsa-miR-181b-5p had the strongest negative correlation with PNCK.

### TF-miRNA-mRNA network

Based on miRwalk3.0 and ENCORI databases, 3438 miRNA-mRNA pairs were obtained. Among them, five mRNAs were related with prognosis of COAD. Then, TF related with hsa-miR-181b-5p was further explored using TransmiR v2.0. Finally, 75 TF-miRNA-mRNA relationships were obtained. In the network, 15 TFs, hsa-miR-181b-5p, and five mRNAs (DMD, PDK4, MSRB3, PALLD, and NTN1) were involved (Fig. 8D).

## Discussion

Although chemotherapy could improve the survival performance of GC patients with peritoneal carcinomatosis, potential ascites tumor markers are still needed to be investigated to improve the limited efficacies for treating GC. In the present study, hsa-miR-181b-5p was confirmed as characteristic miRNAs in GC-associated malignant ascites. The tumor samples were divided into high and low expression groups divided by mean expression levels of hsa-miR-181b-5p, and patients with high hsa-miR-181b-5p expression level had better survival outcomes. In total, 197 mRNAs related with hsa-miR-181b-5p were mainly enriched in muscle activity and vascular smooth muscle contraction. Meanwhile, hsa-miR-181b-5p was positively related with activated CD4 T cells and negatively related with eosinophil. Finally, we proposed that hsa-miR-181b-5p might be benefit for improving the survival of GC by the regulation of muscle activity and the infiltration of CD4 T cells and eosinophil (Supplementary Fig. 2).

MiRNAs are widely expressed in living organisms, hsa-miR-181b-5p was demonstrated as miRNA differentially expressed in GC. Meanwhile, our data showed GC with higher hsa-miR-181b-5p had better survival outcomes. Hsa-miR-181b-5p was significantly down-regulated in Ang II-treated cells, and the plasma levels of miR-181b-5p may serve as novel biomarkers for vascular remodeling [21]. To elucidate the mechanisms associated with hsa-miR-181b-5p responsible for GC development, our data further showed that mRNAs related with hsa-miR-181b-5p were significantly enriched in muscle activity and vascular smooth muscle contraction. In cancer patients, defective skeletal muscle regeneration would lead to muscle wasting, and the progressive muscle wasting was demonstrated as one of main reasons for cancer-related deaths, including GC [22]. Therefore, we speculated that hsa-miR-181b-5p might been proposed as a valuable prognosis biomarker for GC by participating in muscle wasting.

Tumor microenvironment can mediate immune response in various kinds of cancers, and immunotherapy has been focused as a new era for cancer treatment in recent years [23]. Previous evidence showed that tumor infiltrating lymphocytes, such as NK cells, intratumoral T-cell, and CD11c + cells, were related with improved survival performance [24, 25], and CD206 + and CXCL8 + macrophages correlated with poor survival outcomes [26]. Our data showed that hsa-miR-181b-5 was positively related with activated CD4 T cells and negatively related with eosinophil. Similarly, Yuan et al. demonstrated that, as compared with matched paraneoplastic tissue, the ratio of CD4 + T-cells in GC was significantly higher [27]. Furthermore, the critical roles of T cells for elimination and recognition of GC have also been confirmed [28]. Eosinophils are also accepted as one of critical components of the immune microenvironment modulating the progression and initiation of tumor, which are a source of protumorigenic molecules mediated by pro-angiogenic factors and anti-tumorigenic mediated by various kinds of cytokines, including IL-18 and TNF-α [29]. Thus, the benefit of hsa-miR-181b-5p for GC improvement might be mediated by modulating immune regulation, such as CD4 T cells and eosinophil.

Great efforts have been made to improve the diagnosis and treatment of cancers [30–34]. Circulating tumor cells (CTCs) play key roles in the occurrence and metastasis of tumors, and the development of CTC detections may improve the early diagnosis and cancer control [35]. A novel microfluidic device is developed to capture CTCs to detect the residual disease in acute leukemia [36]. It is reported that cancer stem-like cells (CSC) showed rescue effect to nonstem-like cancer cells under radiation therapy and the resistance of CSC was associated with lysosome-mediated autophagy, which help us to understand the mechanism of radiotherapy resistance [37, 38]. Our findings of the biological significance of hsa-miR-181b-5p in GC help us to deeply understand the pathogenesis of GC and the clinical application of hsa-miR-181b-5p as the diagnosis and prognosis biomarker for GC needs a long way to go.

In addition, there are some limitations in the current study. First, the sample size in the discovery dataset GSE126399 was relatively small. Secondly, the control samples in GSE126399 dataset were liver cirrhosis-associated ascites, but not normal peritoneal fluids. Liver cirrhosis may also be associated with the alteration of miRNA and mRNAs expression. Thus, the biological significance of hsa-miR-181b-5p warrants a large amount of validation experiments in vivo and in vitro.

## Conclusion

In conclusion, our data suggest that hsa-miR-181b-5p was aberrantly expressed in GC ascites. hsa-miR-181b-5p showed promising diagnostic value for the risk of GC. Patients with high expression of hsa-miR-181b-5p showed better prognosis than those with low expression. hsa-miR-181b-5p was associated with the regulation of muscle activity and the infiltration of CD4 T cells and eosinophil. The aberrant expression of hsa-miR-181b-5p may be used as the biomarker in clinical practice to predict prognosis and outcome in hospitalized GC patients and facilitate personalized treatment. hsa-miR-181b-5p may influence the survival rate of patients by regulating muscle activity and immune microenvironment. Targeting hsa-miR-181b-5p may be a candidate treatment regimen for GC patients. However, lacking functional verification of hsa-miR-181b-5p in GC by the clinical studies was a limitation. Thus, more prospective studies with larger sample sizes should be designed to confirm the above conclusion.

### Electronic supplementary material

Below is the link to the electronic supplementary material.

Supplementary Material 1

Supplementary Material 2

Supplementary Material 3

## Data Availability

The data used to support the findings of this study are available from the corresponding author upon request.

## Abbreviations

GC: gastric cancer
STAD: stomach adenocarcinoma
RF: Random forest
ROC: receiver operating characteristic
GO: Gene Ontology
BP: biological process
MF: molecular functions
CC: cellular components
KEGG: Kyoto Encyclopedia of Genes and Genomes
WGCNA: weighted gene co expression network analysis
K-M: Kaplan-Meier
TF: Transcription factors
