# Supplementary figures and images for: Ascites-derived hsa-miR-181a-5p serves as a prognostic marker for gastric cancer-associated malignant ascites
Source: BMC Genomics. 2024 Jun 24;25:628. doi: 10.1186/s12864-024-10359-2 (PMC11194912; doi:10.1186/s12864-024-10359-2)

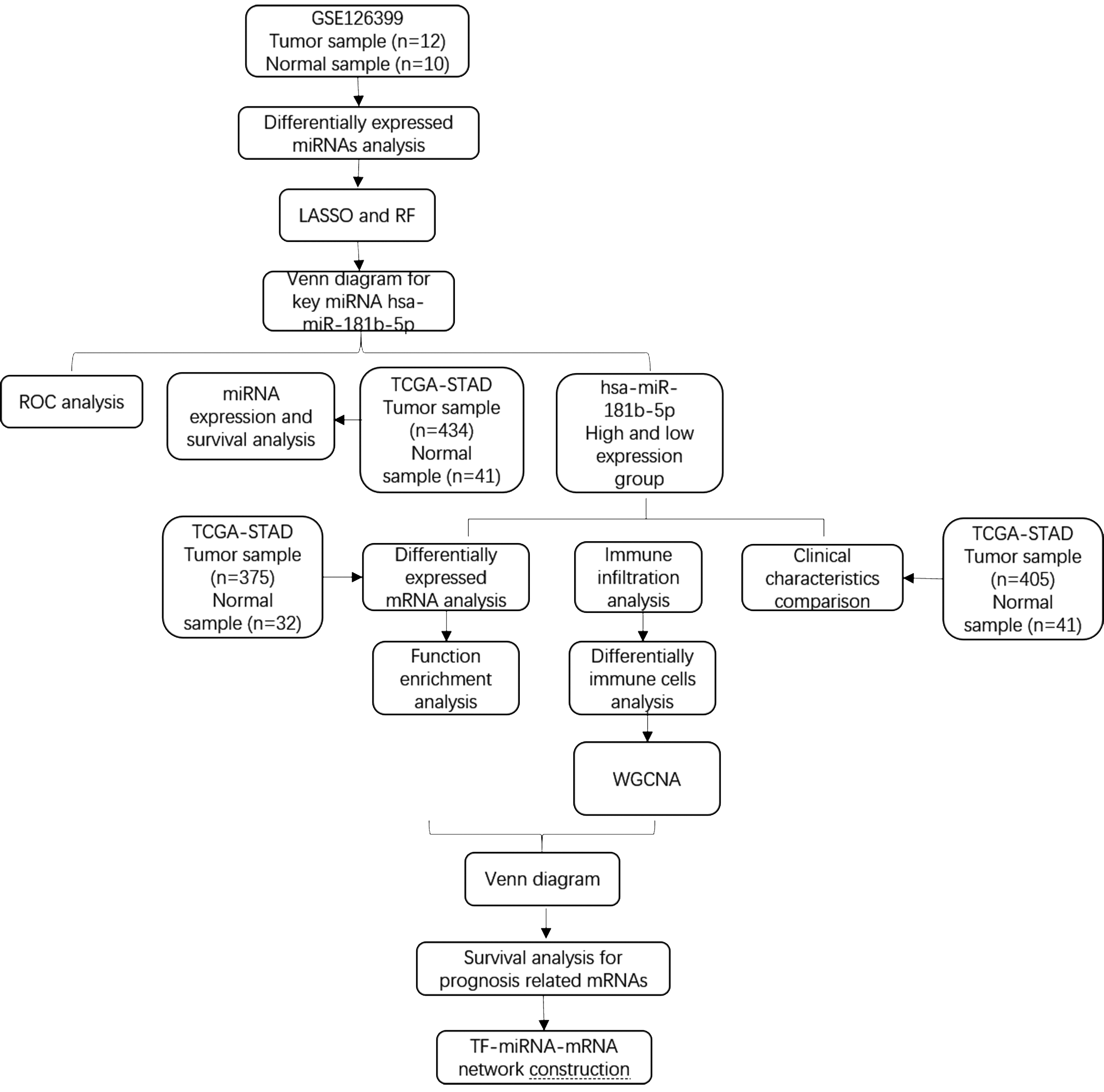

Supplement: Supplementary file 1 — Supplementary Material 1 [file 12864_2024_10359_MOESM1_ESM.tif]

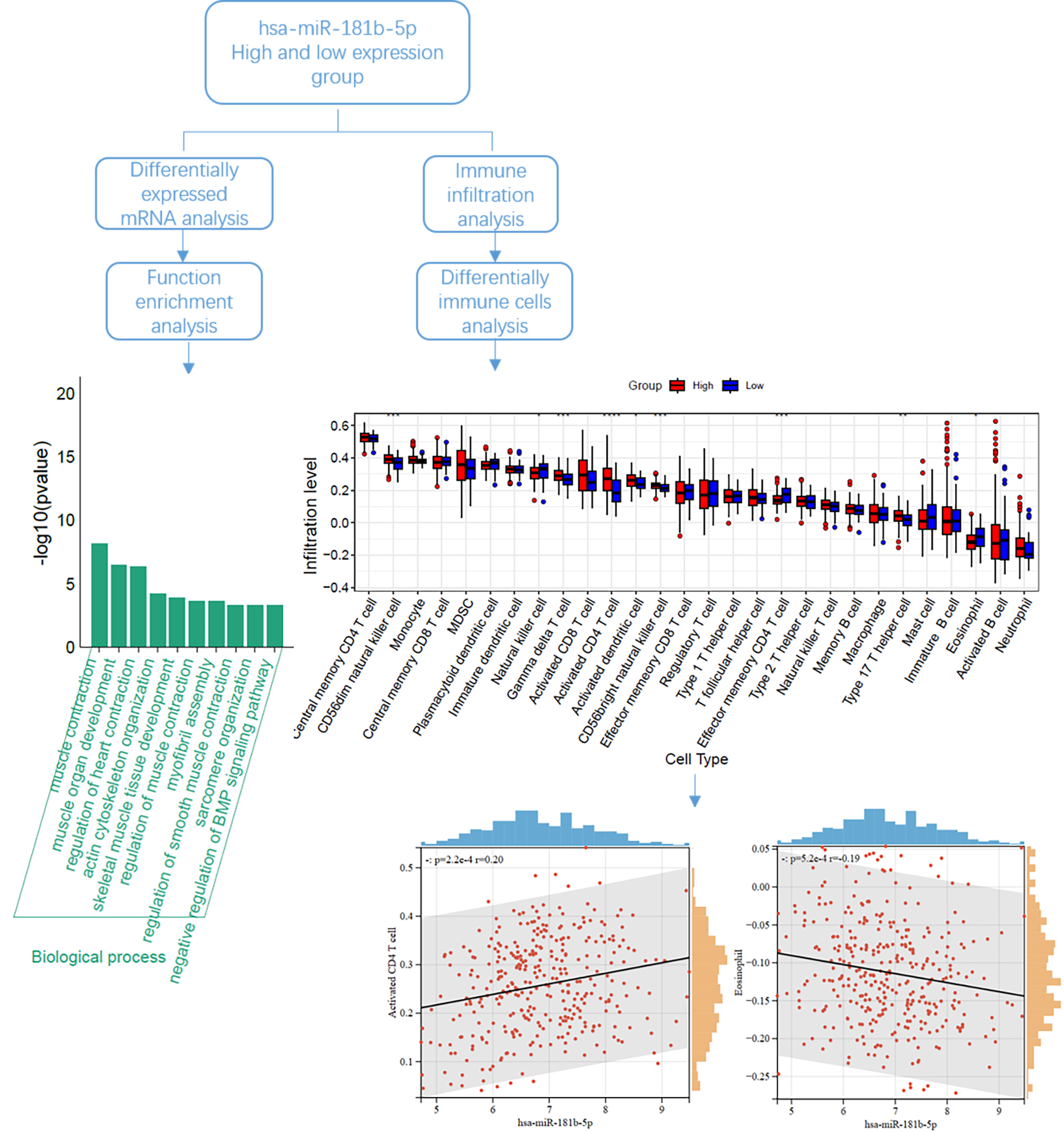

Supplement: Supplementary file 2 — Supplementary Material 2 [file 12864_2024_10359_MOESM2_ESM.tif]
