# Supplementary material for: Ascites-derived hsa-miR-181a-5p serves as a prognostic marker for gastric cancer-associated malignant ascites
Source: BMC Genomics. 2024 Jun 24;25:628. doi: 10.1186/s12864-024-10359-2 (PMC11194912; doi:10.1186/s12864-024-10359-2)
Supplement: Supplementary file 3 — Supplementary Material 3 [file 12864_2024_10359_MOESM3_ESM.doc]

Supplemental Table 1 The basic characteristics in case and control group in GSE126399 dataset

|  | Normal (n=10) | Tumor(n=12) | P value |
| --- | --- | --- | --- |
| Age |  |  | 0.9688 |
| <60 | 4 | 6 |  |
| ≥60 | 6 | 6 |  |
| Gender |  |  | 0.827 |
| Female | 2 | 4 |  |
| Male | 8 | 8 |  |

**Supplemental Table 2 Comparison of basic character**istics in case and control group in TCGA cohort for mRNA expression analysis

|  | Normal(n=32) | Tumor(n=375) | P value |
| --- | --- | --- | --- |
| Age |  |  | 0.1239 |
| <60 | 5 | 112 |  |
| ≥60 | 27 | 259 |  |
| Gender |  |  | 0.7516 |
| Female | 10 | 134 |  |
| Male | 22 | 241 |  |
| Neoplasm_histologic_grade | | | 0.6203 |
| G1 | 0 | 10 |  |
| G2 | 13 | 137 |  |
| G3 | 19 | 219 |  |
| Pathologic_M | |  | 1 |
| M0 | 28 | 330 |  |
| M1 | 2 | 25 |  |
| Pathologic_N | |  | 0.04678 |
| N0 | 12 | 111 |  |
| N1 | 11 | 97 |  |
| N2 | 8 | 75 |  |
| N3 | 0 | 74 |  |
| Pathologic_T | |  | 0.198 |
| T1 | 3 | 19 |  |
| T2 | 11 | 80 |  |
| T3 | 13 | 168 |  |
| T4 | 5 | 100 |  |
| Tumor_stage | |  | 0.08207 |
| stage i | 6 | 53 |  |
| stage ii | 15 | 111 |  |
| stage iii | 6 | 150 |  |
| stage iv | 4 | 38 |  |

**Supplemental Table 3 The basic characteristics of patients and controls in TCGA cohort for miRNA analysis**

|  | Normal (n=41) | Tumor (n=434) | P value |
| --- | --- | --- | --- |
| Age |  |  | 0.2145 |
| <60 | 8 | 129 |  |
| ≥60 | 33 | 300 |  |
| Gender |  |  | 0.7782 |
| female | 16 | 154 |  |
| male | 25 | 280 |  |
| Neoplasm_histologic_grade | |  | 0.5847 |
| G1 | 0 | 10 |  |
| G2 | 16 | 155 |  |
| G3 | 24 | 260 |  |
| Pathologic_M | |  | 0.9048 |
| M0 | 37 | 383 |  |
| M1 | 2 | 29 |  |
| Pathologic_N | |  | 0.01439 |
| N0 | 16 | 129 |  |
| N1 | 13 | 116 |  |
| N2 | 11 | 83 |  |
| N3 | 0 | 87 |  |
| Pathologic_T | |  | 0.05617 |
| T1 | 4 | 23 |  |
| T2 | 15 | 92 |  |
| T3 | 16 | 191 |  |
| T4 | 6 | 118 |  |
| Tumor_stage | |  | 0.02316 |
| stage i | 9 | 58 |  |
| stage ii | 19 | 128 |  |
| stage iii | 8 | 179 |  |
| stage iv | 4 | 42 |  |
